# Overcoming COVID-19 Vaccine Hesitancy: Insights from an Online Population-Based Survey in the United States

**DOI:** 10.3390/vaccines9101100

**Published:** 2021-09-28

**Authors:** Hoda Badr, Xiaotao Zhang, Abiodun Oluyomi, LeChauncy D. Woodard, Omolola E. Adepoju, Syed Ahsan Raza, Christopher I. Amos

**Affiliations:** 1 Department of Medicine, Section of Epidemiology and Population Science, Baylor College of Medicine, Houston, TX 77030, USA; xiaotao.zhang@bcm.edu (X.Z.); abiodun.oluyomi@bcm.edu (A.O.); syed.raza@bcm.edu (S.A.R.); chris.amos@bcm.edu (C.I.A.); 2 Department of Health Systems and Population Health Science, University of Houston College of Medicine, Houston, TX 77004, USA; lwoodard@central.uh.edu (L.D.W.); oadepoju@central.uh.edu (O.E.A.); 3 Humana Integrated Health System Sciences Institute, University of Houston, Houston, TX 77004, USA; 4 Institute of Clinical and Translational Research, Baylor College of Medicine, Houston, TX 77030, USA

**Keywords:** COVID-19, vaccine hesitancy, Health Belief Model, Theory of Planned Behavior

## Abstract

This study sought to identify individual-level determinants of COVID-19 vaccine hesitancy based on the Health Belief Model (HBM) and Theory of Planned Behavior (TPB). An online population-based survey was distributed in English and Spanish. Data were derived from 1208 U.S. adults (52% female; 38.7% minorities), 43.5% of whom reported vaccine hesitancy. Multivariable analysis revealed that unemployed individuals were more likely (OR = 1.78, 95% CI: 1.16–2.73, *p* = 0.009) and married (OR = 0.57, 95% CI: 0.39–0.81, *p* = 0.002) and higher income individuals (OR = 0.52, 95% CI 0.32–0.84, *p* = 0.008) were less likely to be hesitant. Individuals with greater perceived susceptibility to COVID-19 (OR = 0.82, 95% CI: 0.71–0.94, *p* = 0.006), who perceived vaccination as being convenient (OR = 0.86, 95% CI: 0.74–1.00, *p* = 0.047), and who afforded greater importance to cues to action from government (OR = 0.84, 95% CI: 0.74–0.95, *p* = 0.005), public health (OR = 0.70, 95% CI: 0.59–0.82, *p* < 0.001), and healthcare experts (OR = 0.59, 95% CI: 0.50–0.69, *p* < 0.001) were also less likely to be hesitant. Findings suggest that HBM and TPB constructs may be useful in informing strategies to improve COVID-19 vaccine uptake. Specifically, framing appeals based on perceptions of COVID-19 susceptibility, making vaccination convenient, and rebuilding trust through unified cues to action may help to overcome vaccine hesitancy.

## 1. Introduction

As COVID-19 surged in 2020, governments across the globe touted the hope of reaching population-level immunity once vaccines were developed and made available. The thinking at the time was that the pandemic would fade once 60–70% of the population was fully vaccinated [1,2,3]. As of 21 September 2021 we have plenty of reasons to rejoice—more than 5.92 billion vaccine doses have been administered worldwide, and COVID-19 vaccines have been shown to be remarkably safe and effective [4]. However, ten months after the initial vaccine rollout, only 55% of American adults and 32% of adults worldwide are fully vaccinated against COVID-19 [5]. Moreover, a lingering and frustrating impediment to controlling spread of the virus persists—vaccine hesitancy. Vaccine hesitancy refers to either a delay in acceptance or refusal of vaccines [6]. COVID-19 vaccination hesitancy is being fueled by a variety of factors including the novelty of the disease, unusually rapid speed of vaccine development, politicization of the vaccine, and some groups’ mistrust in science and health experts [7,8]. Thanks to new highly transmissible variants like delta that are moving the bar for herd immunity even higher and large proportions of the public that are continuing to decline or postpone vaccination, COVID-19 has evolved into a pandemic of the unvaccinated. Understanding who is more likely to be hesitant and why is therefore imperative so that more targeted interventions to address vaccine hesitancy and improve uptake can be developed.

In a recent online study [9], researchers identified several sociodemographic risk factors for COVID-19 vaccine hesitancy including younger age (18–24 years), non-Asian race, and living in a rural area. Although vaccine hesitancy decreased by one-third from January to May 2021 (with notable decreases among Blacks, Pacific Islanders, and Hispanics, as well as those with lower educational levels), the percentage of those saying they would “definitely not” get vaccinated did not change. This suggests the presence of a highly hesitant group of people who are unlikely to be swayed by traditional information-based approaches for addressing vaccine hesitancy [10]. Researchers also found that almost half of vaccine hesitant responders were afraid of possible effects, and those who were most hesitant were likely to mistrust the vaccine and/or government [9]. These findings support the idea that vaccine hesitancy may be rooted in underlying psychological values. Going beyond exploration of sociodemographic risk factors and developing a clearer understanding of the key psychological drivers of vaccine hesitancy may thus help to develop more targeted and effective vaccine promotion interventions.

Several well-tested theories of health behavior change exist that may be helpful for understanding the range of factors driving COVID-19 vaccine hesitancy and may ultimately be used to catalyze vaccine uptake. Central to these theories is the idea that people engage in an internal decision making process whereby they weigh the pros and cons of getting vaccinated. For example, the Health Belief Model (HBM) [11] proposes that people weigh the severity of the health threat they confront (e.g., perceived susceptibility and severity), and the perceived benefits or harms of taking a specific action (e.g., vaccination) related to that health threat [12]. Their individual risk assessment can be influenced by a variety of factors including cues to action from trusted sources of information, and the social context in which they live and interact [13]. These factors have long been recognized as important predictors of influenza vaccine uptake [14], and emerging research suggests they may also be important for COVID-19 vaccine acceptance [15]. Yet, the HBM has been criticized for neglecting social cognitive and other factors that are recognized by other behavior change models (e.g., Theory of Planned Behavior; TPB) [16] that may be relevant in the context of COVID-19. For example, in the face of widespread disinformation and concerns about equitable access, having accurate knowledge about COVID-19, social expectations (i.e., subjective norms), and the perceived convenience of vaccination (i.e., perceived control) may play a critical role in shaping vaccine intentions. Likewise, sociodemographic variables and personal risk factors that could either modulate susceptibility to COVID-19 (e.g., having a chronic health condition [17]) or influence intentions to vaccinate (e.g., mental health [18], past engagement in other preventive behaviors [19]), are important to consider so that more targeted vaccine promotion messages can be developed. Thus, grounded by the HBM and TPB, this study sought to identify individual-level determinants that are associated with COVID-19 vaccine hesitancy with the goal of informing public health efforts to enhance vaccine acceptance.

## 2. Materials and Methods

### 2.1. Sample and Setting

This study was approved by the Baylor College of Medicine Institutional Review Board H-47505 and is part of a larger cohort study of the psychosocial and behavioral impacts of the COVID-19 pandemic [20]. Eligible individuals were age 18 years or older, fluent in English or Spanish, and resided in the United States.

### 2.2. Data Collection

Surveys were distributed via paid and unpaid social media advertisements and Soapbox Sample, an online survey crowdsourcing platform, between 20 November and 11 December 2020. This window corresponded to the period immediately preceding the initial COVID-19 vaccine rollout in the U.S. Social media advertisements contained a hyperlink that directed individuals to the survey website, where they could review a brief cover letter describing the study. If, after reading the letter, individuals were interested in participating, they were asked to check a box confirming their eligibility, understanding, and consent. The survey was administered online in English and Spanish on the Qualtrics survey platform (Provo, UT, USA) [21].

### 2.3. Measures

Survey items are in Table A1 and described below.

#### 2.3.1. Outcome Variable

Vaccine hesitancy was assessed with the item, “When a government-approved vaccine for COVID-19 becomes available, I will get it,” rated a 5-point Likert-type scale (1 = strongly disagree, 2 = disagree, 3 = unsure, 4 = agree, 5 = strongly agree). The item was aligned with the Community Engagement Alliance (CEAL) Against COVID-19 Disparities common survey questionnaire. Consistent with established definitions of vaccine intention and hesitancy [6], we classified individuals as “hesitant to be vaccinated” if they answered 1, 2, or 3, and “intends to be vaccinated” if they answered 4 or 5.

#### 2.3.2. Individual-Level Determinants

Factors that were examined included sociodemographics, personal risk factors, and social cognitive factors.

##### Sociodemographics

Sociodemographic variables including age, gender, race/ethnicity, marital status, education, annual household income, and work status were assessed.

##### Personal Risk Factors

Measures included physical health status, mental health, and degree of adherence to COVID-19 preventive behaviors.

Physical health status. We asked, “Do you currently have a chronic/serious health condition (yes/no)?” If individuals answered, “yes”, they were asked to specify the condition.

Mental health. The 4-item Patient-Reported Outcome Measure Information System (PROMIS) short form depression measure and the 4-item PROMIS general anxiety measure were administered [22]. For both measures, items were rated from 1 (never) to 5 (always) and scaled into a T-score with a mean of 50 and standard deviation of 10. Scores > 60 indicate significant depression or anxiety symptoms, warranting further psychological evaluation.

COVID-19 preventive behaviors. To assess adherence to social distancing, we asked, “How often do you practice social distancing when you are with others who do not live with you?” Response options were on an 11-point Likert-type scale from 0 = never to 10 = all the time. To assess mask wearing, we asked, “How often do you wear a mask or other face covering when you go out in public?” on a 5-point Likert-type scale from 1= not at all to 5 = all the time. To assess hand hygiene, we asked, “How many times per day do you wash your hands with soap and water for at least 20 s?” and “How many times per day do you use hand sanitizer?” Response options were 1 = 0 times, 2 = 1–3 times, 3 = 4–6 times, and 4 = more than 6 times. The average of these two items was calculated to obtain the hand hygiene score.

##### Social Cognitive Factors

We assessed knowledge and attitudes about COVID-19 and vaccines. To assess knowledge about COVID-19, we used 8 items from previously published studies [23,24,25] (e.g., “COVID-19 is spread through coughing and sneezing”, “People exposed to COVID-10 can spread the disease to others, even if they do not have any symptoms”, “Currently, there is no cure for COVID-19”). Response options were “true”, “false”, and “I don’t know.” A correct answer was assigned 1 point and an incorrect/don’t know answer was assigned 0 points.

To assess vaccine attitudes, 10 items based on the HBM and TPB were derived for this study. Questions were introduced by asking, “How important are each of the following in your decision about whether to get a government-approved COVID-19 vaccine when it becomes available?” Items addressed perceived susceptibility (“My personal risk of getting infected with COVID-19 if I do not take the vaccine”), perceived severity (“How serious the COVID-19 outbreak is in the area where I live”), cues to action (4 items on the role of a national vaccine mandate and recommendations from government representatives, public health experts, and one’s healthcare provider in vaccination decisions), perceived benefit (“Whether the vaccine is free of charge”), perceived barriers (“Whether there are any known serious side effects of the vaccine.”), subjective norms, (“Whether other people I know are being vaccinated”), and perceived control, (“Whether the process for me to be vaccinated is convenient”). All items were rated on a 5-point Likert-type scale (1 = not at all important to 5 = extremely important).

### 2.4. Statistical Analysis

First, for all individual-level determinants, we computed descriptive statistics for the categorical variables (frequency distributions) and continuous variables (mean, standard deviation). Using cross-tabulations, along with Chi-square or independent samples T-tests as appropriate, we assessed whether the proportions of participants that reported each determinant vary significantly between the “hesitant to be vaccinated” and “intends to be vaccinated” groups. Next, unconditional univariate logistic regression models were used to determine bivariable associations between vaccine hesitancy and each of the individual-level determinants assessed. Last, all the variables that had significant bivariable association with vaccine hesitancy in the univariate logistic regression models (95% CI does not contain 1.0) were entered into a single multivariable logistic regression model. The multivariable model produced adjusted odds ratios and 95% confidence intervals (CI). Statistical analyses were performed in SAS V.9.4.

## 3. Results

### 3.1. Sample Characteristics

Overall, of the 1375 individuals that accessed the online survey, 1208 (88%) were included in the final analytic file. The remaining 167 participants were excluded because they did not pass our survey quality control (i.e., ReCaptcha, red herring questions, IP control) and data quality checks (e.g., answer consistency and speed checks). As such, we were unable to compare participants in the analytic file with those who were excluded. No significant differences in reports of vaccine hesitancy were observed based on the sampling method (i.e., paid vs. unpaid social media advertisements).

The median survey completion time was 24.5 min (M = 39.2 min, SD = 1.48 h, Range = 2.85 min to 1.2 days). The sample comprised 628 (52.0%) females, and 467 (38.7%) racial/ethnic minorities. Racial/ethnic minority subgroups included non-Hispanic Blacks (*n* = 208; 17.2%), Hispanics (*n* = 222; 18.4%) and other races (*n* = 37; 3.1%). Average respondent age was 42.6 years (SD = 16.3; Range = 19 to 91 years). Regarding physical health status, 419 (35.1%) respondents had a chronic or serious health condition. The most frequently reported conditions were cancer (*n* = 152; 36.3%) and diabetes (*n* = 117; 27.9%). Regarding mental health, 557 (47.2%) scored above the PROMIS cut-off for depression, and 596 (50.5%) scored above the PROMIS cut-off for anxiety. Respondents reported moderate levels of COVID-19 knowledge (M = 7.3 out of 10, SD = 1.1), low to moderate levels of hand-hygiene (M = 2.8 out of 5, SD = 0.8), moderate to high levels of social distancing (M= 8.1, SD = 2.2), and high levels of mask wearing (M = 4.6 out of 5, SD = 0.8). Six-hundred eighty-two (56.5%) people intended to be vaccinated and 526 (43.5%) were hesitant.

All the individual-level determinants examined, except knowledge, had significantly different proportions (categorical variables) or means (continuous variables) between the two vaccine intention groups (Table 1). Notably, the proportions of participants who reported female gender (59.7%), unmarried status (66.4%), or not college educated (44.7%) were higher among the “hesitant to get vaccinated” group compared with the proportions in the “intends to be vaccinated” group. In contrast, the proportions of participants who reported depression (51.8%), anxiety (54.7%), or a pre-existing health condition (39.1%) were higher among the “intends to be vaccinated” group compared with the proportions in the “hesitant to be vaccinated” group. Also, the mean scores for all the determinants under both preventive behaviors and social cognitive factors were higher among the “intends to be vaccinated” group compared relative to the “hesitant to be vaccinated” group.

### 3.2. Univariate and Multivariable Regression Models

Results from both the univariate and multivariable regression models are in Table 2. In the univariate models, almost all of the sociodemographic and health-related variables showed significant bivariable association with vaccine hesitancy (e.g., non-Hispanic Black race, Hispanic ethnicity, not college educated, pre-existing health condition, anxiety). However, after the sociodemographic and health-related variables were entered into the multivariable logistic regression model, most were no longer significant. In the final model, unemployed individuals were more likely to be hesitant to be vaccinated (OR = 1.78, 95% CI 1.16 to 2.73, *p* = 0.009), whereas married individuals (OR = 0.57, 95% CI 0.39 to 0.81, *p* = 0.002), those with incomes > $75,000 (OR = 0.52, 95% CI 0.32 to 0.84, *p* = 0.008), and those with greater perceived susceptibility to COVID-19 (OR = 0.82, 95% CI 0.71 to 0.94, *p* = 0.006) were less likely to be hesitant. The model also revealed that individuals who perceived the vaccination process as being more convenient (OR = 0.86, 95% CI: 0.74 to 1.00, *p* = 0.047), and afforded greater importance to recommendations from government representatives (OR = 0.84, 95% CI 0.74 to 0.95, *p* = 0.005), public health officials (OR = 0.70, 95% CI 0.59 to 0.82, *p* < 0.001), and healthcare providers (OR = 0.59, 95% CI 0.50 to 0.69, *p* < 0.001) were less likely to be hesitant (Table 2).

## 4. Discussion

This population-based survey sought to identify individual-level determinants of COVID-19 vaccine hesitancy, based on the HBM and TPB. Overall, we found that 43.5% of respondents were hesitant to be vaccinated. Although bivariable analyses identified several sociodemographic and health-related determinants of vaccine hesitancy, once these variables were entered in a multivariable model that included social cognitive variables based on the HBM and TPB, most were no longer significant. The final model showed that whereas unemployed individuals were more likely to be hesitant, married individuals and those with higher incomes and greater perceived susceptibility to COVID-19, who valued expert recommendations and perceived the vaccination process as being more convenient, were less likely to be hesitant. Taken together, these findings underscore the importance of broadening the public health focus beyond sociodemographic and health-related determinants and considering the role that social cognitive factors may play in contributing to COVID-19 vaccine hesitancy [26].

The finding that perceived susceptibility was associated with reduced likelihood of vaccine hesitancy may have important implications for future COVID-19 vaccine promotion campaigns. Scholars have argued that messages that frame the outcome of behavioral decisions as either gains or losses can lead individuals to prefer different courses of action [27]. In fact, in one study, parents who felt their children were at increased risk of contracting HPV were more likely to be persuaded by gain-framed appeals that highlighted benefits of vaccination, whereas parents who felt their children are at low risk were more likely to be persuaded by loss-framed appeals that emphasized the costs of failing to get vaccinated [28]. Together with our findings, this suggests that more targeted COVID-19 vaccine promotion campaigns are needed, and that the effectiveness of differently framed appeals may hinge on perceptions of susceptibility to the virus.

In the current COVID-19 infodemic [29], the public is sorting through an overabundance of vaccine information from a variety of sources—some of which are credible and many of which are not [7]. Lessons learned from previous pandemics (e.g., SARS, MERS) remind us that trusted sources of information and guidance are fundamental to disease control [30], and, in times of high uncertainty, people often look to trustworthy and reliable sources to guide informed decision-making [31,32]. Our findings suggest that members of the public who look to government, scientific, and healthcare experts as trusted sources of information are less likely to be hesitant to be vaccinated. Having a more unified and coordinated public health messaging strategy that is implemented across all levels of government and the scientific and healthcare communities could thus go a long way toward rebuilding public confidence in vaccination—particularly among those subgroups of the population that may be more hesitant due to misinformation, safety concerns stemming from the speed of COVID-19 vaccine development, or historical inequities that are the roots of medical mistrust.

We found that unemployed individuals were more likely to be hesitant to be vaccinated, but that married individuals and those who had higher incomes were less likely to be hesitant. The finding regarding married individuals is consistent with research showing that marital status is associated with better health [33]. Married individuals may also experience greater support, encouragement, and even positive pressure to engage in healthy behaviors, including vaccination [34]. It is also possible that married couples may be more likely to have at least one household member that is employed and/or have higher household incomes [35], which could enhance their access to healthcare and influence perceptions regarding convenience. Similarly, individuals with higher incomes have more healthcare access whereas unemployed individuals have less access.

The finding regarding the importance of public perceptions about the convenience of the vaccination process may have serious implications for the success of ongoing vaccine rollout efforts given that both the Pfizer and Moderna vaccines require two doses, the number of vaccination sites is limited, and nearly 6 in 10 older Americans say they don’t know when or where they can get a COVID-19 vaccine [36]. Indeed, research has shown that issues of convenience can arise due to structural barriers such as difficult access or because attitudes are not strongly against or in favor of vaccination [37]. Under such circumstances, it is important to remove barriers, support self-control (will power), and add incentives [37]. For example, offering the COVID-19 vaccine at local pharmacies and worksites and bundling it with the flu shot may enhance convenience and reduce perceived costs. Likewise, ensuring equitable access by ensuring a sufficient number of conveniently located vaccination locations will be imperative. Finally, campaigns that encourage people to pre-commit (e.g., before their turn) and make a vaccination plan (e.g., when, where) as well as telephone/text messaging reminder campaigns have been effective in supporting self-control (willpower) in other vaccine-preventable diseases [37], and could be similarly effective for COVID-19.

This study has several strengths. First, it is one of the largest COVID-19 vaccine hesitancy studies to date. Second, our use of an online crowdsourcing platform facilitated recruitment of a diverse sample with regard to age, gender, and race/ethnicity. Men and persons aged 65 and over were only slightly underrepresented, non-Hispanic Blacks were slightly overrepresented, and the percentage of Hispanic respondents was generally reflective of the U.S. population. [38] This bolsters generalizability of our study findings—particularly to those racial/ethnic minority groups hit hardest by the pandemic. Third, although other studies have examined correlates and disparities of COVID-19 vaccine hesitancy in the U.S. [39,40,41,42], most were conducted at a time in the pandemic when vaccines were still being developed. Individuals often rely on cognitive biases and heuristics when evaluating hypothetical medical decisions [43], but when they make real decisions, learning and assimilation processes play a more prominent role [44]. This survey was conducted in the weeks immediately preceding FDA emergency use authorization and national vaccine rollout efforts. Although some of the initial hesitancy toward COVID-19 vaccination has waned, time has shown that most individuals who were initially hesitant to get vaccinated have remained steadfast [10]. Thus, our data remain salient because they highlight factors that are relevant to these hesitant individuals and could be incorporated as components of future targeted interventions. Finally, because this study was theoretically grounded, we were able to demonstrate the relative contribution of sociodemographic and social cognitive factors to COVID-19 vaccine hesitancy, which will facilitate development of targeted vaccine messaging campaigns.

This study also has some limitations. First, given the cross-sectional nature of the data, findings represent a snapshot of vaccine hesitancy at a single moment in time. We are unable to account for how attitudes may evolve as the prevalence of COVID-19, availability of vaccines, and political discourse change. Second, there is a potential for social desirability bias, whereby participants may have responded in manner that they believe is viewed favorably by others. Third, we did not assess a variety of benefits and barriers of vaccination. Fourth, our application of HBM and TPB constructs assumes that vaccine uptake is driven by rational decision making. However, for certain population subgroups, the lack of vaccine uptake may be driven by irrational beliefs. In such cases, emotional appeals that attend to negative emotions (e.g., fear, anxiety) and that activate positive emotions (e.g., altruism, patriotism) may be effective. Finally, hesitancy could differ from actual vaccination behavior. It is possible that some individuals who intend to be vaccinated encounter barriers to access that stymie their efforts, whereas others who were initially more hesitant are ultimately persuaded and accept vaccination.

## 5. Conclusions

Overall, our findings suggest the need to tailor future interventions to combat COVID-19 vaccine hesitancy based on the needs of different target populations [37]. For example, for unemployed and low-income individuals who are experiencing financial hardship, vaccine campaigns may need to emphasize that the vaccine is free of charge. For individuals who are more complacent about getting vaccinated, appeals that are framed to increase perceived susceptibility and that emphasize altruistic motives may be effective. For individuals experiencing convenience issues, interventions should aim at eliminating structural barriers and strengthening positive attitudes toward vaccination [45]. Finally, for individuals who lack confidence in the vaccine or government, interventions that seek rebuild public trust through a more unified public health messaging strategy that is adopted across government, scientific, and healthcare communities may go a long way toward overcoming vaccine hesitancy.

## Figures and Tables

**Table 1:** Sample characteristics according to vaccine hesitancy status.

Variables	Total		Vaccine Hesitancy				
			Intends to be Vaccinated (*n* = 682)		Hesitant to be Vaccinated (*n* = 526)		*p*-Value
	*n*	(%)	*n*	(%)	*n*	(%)	
**Age**							<0.001
18–30	352	(29.1)	166	(24.3)	186	(35.4)	
31–50	498	(41.2)	311	(45.6)	187	(35.6)	
51–65	205	(17.0)	102	(15.0)	103	(19.6)	
>65	153	(12.7)	103	(15.1)	50	(9.5)	
**Gender**							<0.001
Female	628	(52.0)	314	(46.0)	314	(59.7)	
Male	580	(48.0)	368	(54.0)	212	(40.3)	
**Race/Ethnicity**							<0.001
non-Hispanic White	741	(61.3)	454	(66.6)	287	(54.6)	
non-Hispanic Black	208	(17.2)	89	(13.1)	119	(22.6)	
Hispanic	222	(18.4)	118	(17.3)	104	(19.8)	
Other	37	(3.1)	21	(3.1)	16	(3.0)	
**Marital status**							<0.001
Unmarried	619	(51.3)	270	(39.7)	349	(66.4)	
Married	588	(48.7)	411	(60.4)	177	(33.7)	
**Education**							<0.001
Not college educated	379	(31.4)	144	(21.1)	235	(44.7)	
College educated	829	(68.6)	538	(78.9)	291	(55.3)	
**Income**							<0.001
Less than $25,000	291	(24.1)	114	(16.7)	177	(33.7)	
$25,000 to $74,999	438	(36.3)	217	(31.9)	221	(42.1)	
$75,000 or more	477	(39.6)	350	(51.4)	127	(24.2)	
**Work status**							<0.001
Working full time	615	(51.0)	400	(58.7)	215	(41.0)	
Working part time	167	(13.9)	85	(12.5)	82	(15.6)	
Retired	156	(12.9)	99	(14.5)	57	(10.9)	
Unemployed	268	(22.2)	97	(14.2)	171	(32.6)	
Mental Health: Depression *							<0.001
Yes	557	(47.2)	348	(51.8)	209	(41.2)	
No	622	(52.8)	324	(48.2)	298	(58.8)	
Mental Health: Anxiety *							0.001
Yes	596	(50.5)	366	(54.7)	230	(45.1)	
No	583	(49.5)	303	(45.3)	280	(54.9)	
**Physical Health Status: Pre-existing health condition**							
Yes	419	(35.1)	263	(39.1)	156	(29.9)	0.001
No	774	(64.9)	409	(60.9)	365	(70.1)	
	**Mean**	**(SD)**	**Mean**	**(SD)**	**Mean**	**(SD)**	
**COVID-19 preventive behaviors**							
Social distancing	8.1	(2.2)	8.5	(1.7)	7.5	(2.6)	<0.001
Wear mask/face covering in public	4.6	(0.9)	4.7	(0.7)	4.4	(1.0)	<0.001
Hand hygiene	2.8	(0.8)	2.9	(0.7)	2.7	(0.8)	<0.001
**Social cognitive factors**							
COVID-19 knowledge score	7.3	(1.1)	7.3	(1.0)	7.3	(1.2)	>0.99
Perceived susceptibility	3.6	(1.4)	4.0	(1.1)	3.0	(1.4)	<0.001
Perceived severity	3.4	(1.4)	3.7	(1.3)	3.0	(1.5)	<0.001
Cues to action (national vaccine mandate)	3.3	(1.5)	3.6	(1.4)	2.9	(1.5)	<0.001
Cues to action (government representatives)	2.8	(1.5)	3.3	(1.5)	2.2	(1.4)	<0.001
Cues to action (public health experts)	3.5	(1.4)	4.1	(1.0)	2.7	(1.4)	<0.001
Cues to action (one’s healthcare provider)	3.6	(1.3)	4.1	(1.0)	2.8	(1.2)	<0.001
Perceived benefits	3.3	(1.5)	3.6	(1.4)	2.9	(1.5)	<0.001
Perceived barriers	3.9	(1.3)	4.2	(1.0)	3.6	(1.5)	<0.001
Subjective norms	2.8	(1.5)	3.2	(1.3)	2.3	(1.4)	<0.001
Perceived control (convenience)	3.2	(1.5)	3.6	(1.4)	2.7	(1.5)	<0.001

NOTE: Missing data not included in statistical analyses. * Individuals categorized as having depression or anxiety met the criteria for caseness (T-score > 60) on the PROMIS 4-item short-form depression and anxiety measures.

**Table 2:** Univariate and multivariable analysis for vaccine hesitancy *.

Variables	Crude OR								MV adjusted OR ^†^					
	OR		95% CI				*p* Value		OR		95% CI			*p*-Value
**Age**														
18–30	Ref								Ref.					
31–50	0.54		0.41−0.71				<0.001		1.26		0.83−1.90			0.28
51–65	0.90		0.64−1.27				0.55		1.37		0.82−2.30			0.23
>65	0.43		0.29−0.65				<0.001		0.99		0.46−2.15			0.98
**Gender**														
Female	Ref								Ref.					
Male	0.58		0.46−0.73				<0.001		0.72		0.52−1.01			0.06
**Race/Ethnicity**														
White	Ref								Ref.					
Black	2.12		1.55−2.89				<0.001		1.15		0.75−1.75			0.53
Hispanic	1.39		1.03−1.89				0.03		0.94		0.61−1.43			0.76
Other	1.21		0.62−2.35				0.58		0.98		0.39−2.49			0.97
**Marital status**														
Unmarried	Ref								Ref.					
Married	0.33		0.26−0.42				<0.001		0.57		0.39−0.81			0.002
**Education**														
Not college educated	Ref								Ref.					
College educated	0.33		0.26−0.43				<0.001		0.70		0.49−1.004			0.052
**Income**														
Less than $25,000	Ref								Ref					
$25,000 to $74,999	0.66		0.49−0.89				0.006		0.95		0.63−1.44			0.81
$75,000 or more	0.23		0.17−0.32				<0.001		0.52		0.32−0.84			0.008
**Work status**														
Working full time	Ref.								Ref.					
Working part time	1.80		1.27−1.54				<0.001		1.31		0.81−2.12			0.28
Retired	1.07		0.74−1.54				0.71		0.94		0.47−1.89			0.87
Unemployed	3.28		2.43−4.42				0.001		1.78		1.16−2.73			0.009
**Mental Health: Depression ^‡^**														
No	Ref.								Ref.					
Yes	0.65		0.52−0.82				<0.001		0.75		0.49−1.16			0.20
**Mental Health: Anxiety ^‡^**														
No	Ref.								Ref.					
Yes	0.68		0.54−0.86				0.001		0.91		0.59−1.40			0.67
**Physical Health Status: Pre-existing health condition**														
No	Ref.								Ref.					
Yes	0.67		0.52−0.85				0.001		0.73		0.51−1.03			0.07
**COVID-19 preventive behaviors (1-unit increase**														−
Social distancing	0.81		0.76−0.85				<0.001		0.96		0.88−1.05			0.41
Wear mask/face covering in public	0.72		0.63−0.82				<0.001		1.05		0.84−1.30			0.69
Hand hygiene	0.76		0.65−0.88				<0.001		1.12		0.90−1.41			0.32
**Social cognitive factors (1-unit increase)**														−
COVID-19 knowledge score	1.00		0.87−1.15				>0.99					−		
Perceived susceptibility	0.54		0.49−0.60				<0.001		0.82		0.71−0.94			0.006
Perceived severity	0.68		0.63−0.74				<0.001		1.03		0.90−1.19			0.64
Cues to action (national vaccine mandate)	0.70		0.65−0.76				<0.001		1.08		0.94−1.24			0.31
Cues to action (government official)	0.59		0.55−0.65				<0.001		0.84		0.74−0.95			0.005
Cues to action (public health officials)	0.42		0.38−0.47				<0.001		0.70		0.59−0.82			<0.001
Cues to action (one’s healthcare provider)	0.37		0.33−0.42				<0.001		0.59		0.50−0.69			<0.001
Perceived benefits	0.74		0.68−0.80				<0.001		0.97		0.84−1.10			0.60
Perceived barriers	0.69		0.63−0.76				<0.001		1.11		0.95−1.29			0.19
Subjective norms	0.67		0.62−0.73				<0.001		1.04		0.91−1.20			0.56
Perceived control (convenience)	0.67		0.62−0.72				<0.001		0.86		0.74−1.00			0.047

NOTE: Missing data not included in statistical analyses. * In the analysis, outcome is “hesitant to get vaccinated”. ^†^ These factors that were significantly associated with vaccine hesitancy in univariate logistic regression analysis (95% CI does not contain 1.0), were included into final multivariable logistic regression. ^‡^ Individuals categorized as having depression or anxiety met the criteria for caseness (T-score > 60) on the PROMIS 4-item short-form depression and anxiety measures.

## Data Availability

The datasets generated during and/or analyzed during the current study are available from the corresponding author on reasonable request.

## Appendix A

**Table A1:** Survey measures.

Construct	Measure
**Outcome Variable**	
Vaccine Hesitancy	“When a government-approved vaccine for COVID-19 becomes available, I will get it,” rated a 5-point Likert-type scale (1 = strongly disagree, 2 = disagree, 3 = unsure, 4 = agree, 5 = strongly agree). We classified individuals as “hesitant to be vaccinated” if they answered 1, 2, or 3, and “intends to be vaccinated” if they answered 4 or 5, consistent with established definitions of vaccine intention and hesitancy [6].
**Individual Level Determinants**	
Sociodemographics (7 items)	Age, gender, race/ethnicity, education, marital status, annual household income, work status.
**Personal Risk Factors**	
Physical Health Status	“Do you currently have a chronic/serious health condition (yes/no)?” If “yes”, please specify.
**Mental Health**	
General Depression (4 items)	PROMIS Depression 4-item Short form [22]
General Anxiety (4 items)	PROMIS Anxiety 4-item Short Form [22]
**COVID-19 Preventive Measures**	
Social Distancing	“How often do you practice social distancing when you are with others who do not live with you?” (0 = never to 10 = all the time)
Mask Wearing	“How often do you wear a mask or other face covering when you go out in public?” (1 = never to 5 = all the time)
Hand Hygiene	“How many times per day do you wash your hands with soap and water for at least 20 s?” and “How many times per day do you use hand sanitizer?” (1 = 0 times, 2 = 1–3 times, 3 = 4–6 times and 4 = >6 times)
**Social Cognitive Factors**	
Knowledge	8 items adapted from previously published studies [26,27,28] (e.g., “COVID-19 is spread through coughing and sneezing”, “People exposed to COVID-10 can spread the disease to others, even if they do not have any symptoms”, “Currently, there is no cure for COVID-19”). Response options were “true”, “false”, and “I don’t know.”
Attitudes	Ten items based on the HBM and TPB tapping vaccine attitudes were derived for this study. Questions were introduced by asking, “How important are each of the following in your decision about whether to get a government-approved COVID-19 vaccine when it becomes available?” All items were rated on a 5-point Likert-type scale (1 = not at all important to 5 = extremely important).
Perceived Susceptibility	“My personal risk of getting infected with COVID-19 if I do not take the vaccine.”
Perceived Severity	“How serious the COVID-19 outbreak is in the area where I live”
Cues to Action	4 items on the role of a national vaccine mandate and recommendations from government representatives, public health experts, and one’s healthcare provider in vaccination decisions.
Perceived Benefit	“Whether the vaccine is free of charge.”
Perceived Barriers	“Whether there are any known serious side effects of the vaccine.”
Subjective Norms	“Whether other people I know are being vaccinated.”
Perceived Control (Convenience)	“Whether the process for me to be vaccinated is convenient.”
